# Molecular mechanism of ALKBH5‐mediated m6A demethylation regulating lipopolysaccharide‐induced epithelial–mesenchymal transition in sepsis‐induced acute kidney injury

**DOI:** 10.1002/kjm2.12892

**Published:** 2024-09-17

**Authors:** Hai‐Hong Zhao, Chun‐Ling Chen, Fen‐Fang Chen, Lu‐Lu Zhang, Mei‐Mei Li, Ze‐Bao He

**Affiliations:** ^1^ Department of Infectious Diseases Taizhou Hospital of Zhejiang Province affiliated to Wenzhou Medical University Taizhou China; ^2^ Department of Infectious Diseases Enze Hospital, Taizhou Enze Medical Center (Group) Taizhou China; ^3^ Department of Anesthesiology Taizhou Hospital of Zhejiang Province affiliated to Wenzhou Medical University Taizhou China

**Keywords:** ALKBH5, DXX5, epithelial–mesenchymal transition, miR‐205‐5p, sepsis‐associated acute kidney injury

## Abstract

This study explored the mechanism by which the m6A demethylase ALKBH5 mediates epithelial–mesenchymal transition (EMT) in sepsis‐associated acute kidney injury (SA‐AKI) and AKI‐chronic kidney disease (CKD) transition. HK‐2 cells were stimulated with lipopolysaccharide (LPS) to establish an in vitro model of SA‐AKI. ALKBH5 expression was reduced through the transfection of si‐ALKBH5. Cell viability, apoptosis, and migration were detected by CCK‐8 assay, TUNEL staining, and Transwell. The levels of TNF‐α, IL‐1β, and IL‐6 were measured by enzyme‐linked immunosorbent assay. Quantitative real‐time polymerase chain reaction or Western blotting was performed to determine the expressions of ALKBH5, miR‐205‐5p, DDX5, E‐cadherin, and α‐SMA. The m6A level was quantitatively analyzed. The expression of pri‐miR‐205 bound to DGCR8 and m6A‐modified pri‐miR‐205 after intervention with ALKBH5 expression was detected by RNA immunoprecipitation. A dual‐luciferase assay confirmed the binding between miR‐205‐5p and DDX5. ALKBH5 was highly expressed in LPS‐induced HK‐2 cells. Inhibition of ALKBH5 increased cell viability, repressed apoptosis, and reduced EMT. Inhibition of ALKBH5 increased the m6A modification level, thereby promoting DGCR8 binding to pri‐miR‐205 to increase miR‐205‐5p expression and eventually targeting DDX5 expression. Low expression of miR‐205‐5p or overexpression of DDX5 partially abolished the inhibitory effect of ALKBH5 silencing on EMT. In conclusion, ALKBH5 represses miR‐205‐5p expression by removing m6A modification to upregulate DDX5 expression, thereby promoting EMT and AKI‐CKD transition after SA‐AKI.

## 1 INTRODUCTION

Sepsis is a fatal organ dysfunction that occurs when the host has an unregulated immune response to overwhelming infection. One of the most vulnerable organs affected is the kidneys, which is known as sepsis‐associated acute kidney injury (SA‐AKI), resulting in undesirable morbidity and mortality.^1^ AKI contributes to the development and progression of chronic kidney disease (CKD).^2^ After the initial injury, the surviving tubular epithelial cells undergo dedifferentiation for proliferation and kidney repair. During this process, normal repair can restore tubular epithelial integrity and function, whereas incomplete or maladaptive repair can lead to the development of chronic pathologies such as interstitial fibrosis and the progression to CKD.^3^ The primary intervention target for SA‐AKI is to retard fibrosis (maladaptive repair) while facilitating regeneration (viable epithelial cell proliferation).^4^ Renal fibrosis is a common pathway for almost all forms of renal disease and manifests as hyperplasia of interstitial cells, excessive synthesis of the intercellular matrix, activation of fibroblasts, and imbalance in extracellular matrix secretion and degradation, leading to glomerulosclerosis and renal tubulointerstitial fibrosis.^5^ In particular, the process of endothelial‐to‐mesenchymal transition (EMT), which involves the phenotypic transition of endothelial cells to obtain fully mesenchymal properties, contributes to the development of renal fibrosis and subsequent acute/chronic kidney injury.^6^ An increasing number of studies have confirmed that the profibrotic phenotype transformation of renal tubular epithelial cells, which is characterized mainly by EMT and profibrotic factor secretion, is the direct cause of AKI developing into CKD.^3^ Hence, further elucidation of the mechanism mediating EMT is necessary to develop more effective intervention targets for SA‐AKI.

N6‐methyladenosine (m6A) modification, the most prevalent type of RNA modification in eukaryotes, has gained widespread research interest, particularly in relation to its role in various organ dysfunctions induced by sepsis, including AKI.^7^, ^8^, ^9^ Typically, m6A methylation is performed by methyltransferases (such as METTL3/METTL14/WTAP) and removed by demethylases (such as FTO and ALKBH5). In particular, the m6A demethylase AlkB homolog 5 (ALKBH5) has been demonstrated to play a functional role in renal fibrosis. For example, conditional knockdown of ALKBH5 represses fibrosis of tubular epithelial cells and relieves ischemia/reperfusion‐induced AKI.^10^ Inhibition of ALKBH5 also curbs the malignant biological behaviors of HK‐2 cells stimulated with LPS in vitro, suggesting the potential of ALKBH5 as a target for the prevention and treatment of SA‐AKI.^11^ ALKBH5‐mediated m6A demethylation may be critical in the pathophysiological process of EMT and may even be a therapeutic target for SA‐AKI.

microRNAs (miRNAs) are small noncoding RNAs harboring 18–22 nts that function in the posttranscriptional regulation of gene expression by recognizing the 3′ UTRs of target genes.^12^ Many miRNAs have been confirmed to mediate the pathological processes of SA‐AKI.^13^ An increasing number of studies have reported the critical role of miRNAs in organ fibrosis through their ability to mediate EMT.^14^ One such miRNA, miR‐205‐5p, can functionally restrain SA‐AKI development, as evidenced by the repression of LPS‐stimulated HK2 cell proliferation, inflammation, and oxidative stress.^15^ miR‐205‐5p is weakly expressed in renal carcinoma cells, and miR‐205‐5p overexpression accelerates apoptosis but suppresses EMT in renal carcinoma cells.^16^ On the basis of the above findings, we speculated that ALKBH5‐mediated m6A demethylation promotes EMT and exacerbates SA‐AKI injury by repressing miR‐205‐5p expression. The present study investigated the molecular mechanism by which ALKBH5‐mediated m6A modification regulates LPS‐induced EMT in SA‐AKI and AKI‐CKD, thus providing a theoretical basis for the management of SA‐AKI.

## 2 MATERIALS AND METHODS

### 2.1 Cell culture and establishment of a cell model of SA‐AKI

Human renal cortical proximal convoluted tubular epithelial cells (HK‐2) (CL‐0109, Procell, Wuhan, China) were cultured in Dulbecco's modified Eagle's medium (PM150210, Procell) supplemented with 10% fetal bovine serum (FBS; 10099158, Gibco, Carlsbad, CA, USA) and 1% penicillin/streptomycin (15140148, Gibco) at 37°C with 5% CO2.

To establish a cell model of SA‐AKI, HK‐2 cells were seeded into 96‐well plates (5 × 10^5^ cells/well) and incubated with lipopolysaccharide (LPS; 10 μg/mL, L6386, Sigma, St. Louis, USA) for 24 h after reaching 80% confluence. The cells cultured under conventional conditions were used as the control group.

### 2.2 Cell transfection

siRNAs targeting ALKBH5 (si‐ALKBH5‐1, si‐ALKBH5‐2) and its negative control (si‐NC), the miR‐205‐5p inhibitor and its negative control (inhibitor NC), the miR‐205‐5p mimic and its negative control (mimic NC), the DEAD‐box protein 5 (DDX5) overexpression plasmid (oe‐DDX5) and its empty control (oe‐NC) were all provided by GenePharma (Shanghai, China). The above RNA or vector was transfected into cells via Lipofectamine 2000 (Invitrogen, Carlsbad, CA, USA). After 48 h, the cells were harvested to detect the transfection efficiency and establish the SA‐AKI model.

### 2.3 Cell counting kit‐8 (CCK‐8) assay

HK‐2 cells were seeded into 96‐well plates (5 × 10^3^ cells/well). After 48 h, the cells were treated with 10 μL of CCK‐8 reagent (CK04, Dojindo Laboratories, Japan) for 3 h. The absorbance at 450 nm was measured with a microplate reader (Bio‐Rad, Hercules, CA, USA).

### 2.4 Terminal deoxynucleotidyl transferase (TdT) dUTP nick‐end labeling (TUNEL)

HK‐2 cells (1 × 10^6^) were resuspended in 0.5 mL of phosphate‐buffered saline (PBS), and apoptosis was evaluated via a TUNEL assay kit (ab66108, Abcam, Cambridge, MA, USA). The cells were imaged under a fluorescence microscope (LSM700b, Zeiss, Oberkochen, Germany).

### 2.5 Transwell

The cells were subsequently centrifuged, resuspended in FBS‐free medium, and counted. A cell suspension containing 2 × 10^5^ cells was added to the Transwell apical chamber (Corning, NY, USA), and complete medium containing 10% FBS was added to the basolateral chamber. After 24 h, the cells were fixed with 4% methanol and then stained with 0.1% crystal violet. The cells were imaged and counted with ImageJ software (NIH, Bethesda, Maryland, USA).

### 2.6 Enzyme‐linked immunosorbent assay (ELISA)

The contents of TNF‐α, IL‐1β, and IL‐6 in the cells were tested via a TNF‐α ELISA kit (ab181421, Abcam), an IL‐1β ELISA kit (ab214025, Abcam), and an IL‐6 ELISA kit (ab178013, Abcam).

### 2.7 Quantitative real‐time polymerase chain reaction (qRT‐PCR)

Total RNA was extracted via TRIzol reagent (15596026CN, Invitrogen) and reverse transcribed into cDNA via a RevertAid First Strand cDNA Synthesis Kit (K1622, Thermo Scientific, Waltham, MA, USA). A SYBR Green Quantitative Kit was used for qPCR. miRNA quantification was performed via a TaqMan microRNA Assay Kit (4427975, Thermo Scientific), with glyceraldehyde‐3‐phosphate dehydrogenase (GAPDH) as the internal reference of mRNA and U6 as the internal reference of miRNA.^17^ The relative expression of RNA was calculated by the 2^−ΔΔCt^ method.^18^ The primer sequences are shown in Table 1.

**TABLE 1:** qPCR primers.

	Forward primer (5′–3′)	Reverse primer (5′–3′)
ALKBH5	ACGGATCCTGGAGATGGACA	ATCTTCACCTTTCGGGCAGG
miR‐205‐5p	CTTGTCCTTCATTCCACCGGA	TGCCGCCTGAACTTCACTCC
pri‐miR‐205	TATGGCACACACAGTGGGAC	GTTCAACACGTCCTTGCACC
DDX5	CAAGAGCGTGACTGGGTTCT	CTCCTCTACCCCTGGAACGA
GAPDH	GATTCCACCCATGGCAAATTC	CTGGAAGATGGTGATGGGATT
U6	CTCGCTTCGGCAGCACA	AACGCTTCACGAATTTGCGT

Abbreviations: ALKBH5, AlkB homolog 5; DDX5, DEAD‐box protein 5; GAPDH, glyceraldehyde‐3‐phosphate dehydrogenase; miR‐205‐5p, microRNA‐205‐5p.

### 2.8 Western blot

The cells were lysed with radioimmunoprecipitation assay buffer (Solarbio, Beijing, China), and the protein concentration was determined by the bicinchoninic acid method (Beyotime). The proteins were separated by 12% SDS–PAGE and transferred onto polyvinylidene fluoride membranes (Millipore, Billerica, MA, USA). The membranes were blocked with 5% skim milk for 2 h and incubated with primary antibodies. After being washed three times with Tris‐buffered saline, the membranes were incubated with the secondary antibody IgG (1:2000, ab205718, Abcam) for 2 h at room temperature. The primary antibodies used included ALKBH5 (1:1000, ab195377, Abcam), E‐cadherin (1:1000, ab314063, Abcam), α‐SMA (1:1000, ab314895, Abcam), DDX5 (1:20000, ab126730, Abcam), and β‐tubulin (1:2000, ab52623, Abcam). The protein bands were visualized via SuperSignal West Pico PLUS chemiluminescent substrate (34580, Thermo) and Quantity One software (Bio‐Rad), with β‐tubulin as the internal reference.

### 2.9 Detection of the total m6A level

Total cellular RNA was extracted, and the m6A level in total RNA was determined via an m6A RNA methylation quantification kit (ab185912; Abcam). Briefly, 200 ng of RNA and 80 μL of binding solution were added to 96‐well plates and incubated at 37°C for 90 min for RNA binding. Then, 50 μL of capture antibody was added to each well and incubated for 60 min at room temperature. Subsequently, the detection antibody and enhancer solution were added to each well and incubated at room temperature for 30 min. Finally, the developer solution was supplemented and incubated at 25°C in the dark for 5 min, followed by the addition of stop solution. The absorbance was measured at 450 nm with a microplate reader. The percentage of m6A in total RNA was calculated according to the following formula:

$$m6A\%=\frac{\left(\text{Sample}\;\mathrm{OD}-\mathrm{NC}\;\mathrm{OD}\right)/S}{\left(\mathrm{PC}\;\mathrm{OD}-\mathrm{NC}\;\mathrm{OD}\right)}\times 100\%,$$

where NC and PC are the negative control and positive control provided in the kit, respectively, *S* the amount of RNA input, and *P* is the amount of RNA of the positive control, and the unit is ng.

### 2.10 RNA immunoprecipitation (RIP)

Total RNA was extracted from the cells. m6A antibody (1:50, ab208577, Abcam), DGCR8 antibody (1:60, ab191875, Abcam), or IgG antibody (1:50, ab172730, Abcam) was coupled with protein A/G magnetic beads and placed in IP buffer (140 nM NaCl, 1% NP‐40, 2 mM EDTA, and 20 mM Tris pH 7.5) at 4°C overnight. The immunoprecipitated RNA was eluted and reverse transcribed for qRT‐PCR to measure the level of pri‐miR‐205. Table 1 shows the primers used.

### 2.11 Dual‐luciferase reporter gene assay

The synthesized DDX5 3′‐UTR gene fragment DDX5‐WT containing the binding site with miR‐205‐5p and DDX5‐MUT mutated according to the binding site was constructed into the pMIR‐reporter plasmid (AM5795, Thermo Scientific). The constructed luciferase reporter plasmid was cotransfected into HK‐2 cells with mimic NC or the miR‐205‐5p mimic. After 48 h of transfection, the cells were collected and lysed. Luciferase activity was detected via a luciferase assay kit (K801‐200, BioVision, Mountain View, CA, USA).

### 2.12 Bioinformatics

The downstream target genes of miR‐205‐5p were predicted via StarBase^19^ (https://rnasysu.com/encori/), TargetScan^20^ (https://www.targetscan.org/vert_80/), miRDB^21^ (https://mirdb.org/), and miRTarBase^22^ (https://mirtarbase.cuhk.edu.cn/).

### 2.13 Statistical analysis

Data analysis and map plotting were performed via SPSS 21.0 (IBM Corp., Armonk, NY, USA) and GraphPad Prism 8.0 (GraphPad Software Inc., San Diego, CA, USA). The data were examined for a normal distribution and homogeneity of variance. A *t*‐test was used for comparisons between two groups, and one‐way or two‐way analysis of variance (ANOVA) was used for comparisons among multiple groups, followed by Tukey's multiple comparison test. A value of *p* < 0.05 indicated a significant difference.

## 3 RESULTS

### 3.1 Inhibition of ALKBH5 alleviates LPS‐induced EMT

**FIGURE 1:**
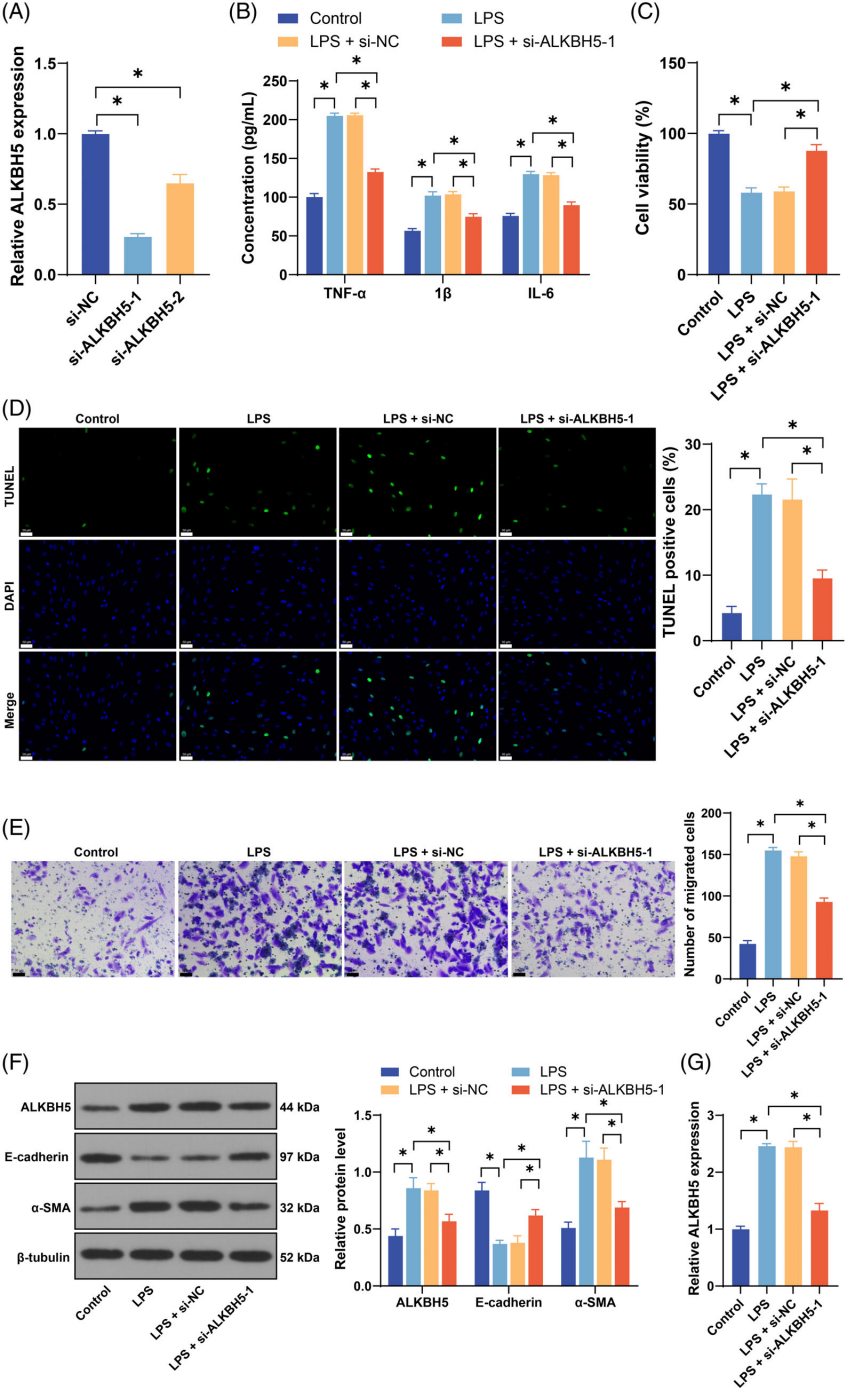
Inhibition of ALKBH5 alleviates LPS‐induced EMT. siRNAs targeting ALKBH5 (si‐ALKBH5‐1, si‐ALKBH5‐2) were transfected into HK‐2 cells, with si‐NC as a control. (A) Transfection efficiency was confirmed by qRT‐PCR. Then, the cells were treated with 10 μg/mL LPS. (B) The levels of TNF‐α, IL‐1β, and IL‐6 in cells were detected via ELISA. (C) Cell viability was measured by a CCK‐8 assay. (D) Cell apoptosis was evaluated by TUNEL staining. (E) Cell migration was measured via Transwell assays. (F) The protein levels of ALKBH5, E‐cadherin, and α‐SMA were detected by Western blot. (G) ALKBH5 expression was detected via qRT‐PCR. The cell experiments were independently repeated three times. The data are presented as the mean ± SD. The data in panels A, C–E, and G were analyzed via one‐way ANOVA, and the data in panels B and F were analyzed via two‐way ANOVA, followed by Tukey's multiple comparisons test. *p < 0.05. ALKBH5, AlkB homolog 5; ELISA, enzyme‐linked immunosorbent assay; EMT, epithelial–mesenchymal transition; LPS, lipopolysaccharide; NC, negative control; qRT‐PCR, quantitative real‐time polymerase chain reaction.

ALKBH5 expression is increased in SA‐AKI.^23^ To explore the effect of ALKBH5 on LPS‐induced EMT in SA‐AKI, we silenced ALKBH5 expression in cells (*p* < 0.05, Figure 1A,F) and then selected si‐ALKBH5‐1, which has better transfection efficiency, for subsequent experiments. LPS treatment dramatically increased proinflammatory factor levels (*p* < 0.05, Figure 1B), reduced cell viability (*p* < 0.05, Figure 1C), increased apoptosis (*p* < 0.05, Figure 1D), enhanced cell migration (*p* < 0.05, Figure 1E), decreased E‐cadherin protein levels, and elevated α‐SMA protein levels (*p* < 0.05, Figure 1F), indicating that EMT occurred in cells. Moreover, LPS induced the upregulation of ALKBH5 expression (*p* < 0.05, Figure 1F,G). The silencing of ALKBH5 (*p* < 0.05, Figure 1F,G) led to decreased levels of proinflammatory factors (*p* < 0.05, Figure 1B), increased cell viability (*p* < 0.05, Figure 1C), weakened apoptosis (*p* < 0.05, Figure 1D) and migration (*p* < 0.05, Figure 1E), increased E‐cadherin protein levels, and decreased α‐SMA protein levels (*p* < 0.05, Figure 1F), indicating that the inhibition of ALKBH5 effectively alleviated LPS‐induced EMT.

### 3.2 ALKBH5 inhibits the processing of pri‐miR‐205 into miR‐205‐5p by removing m6A modifications

**FIGURE 2:**
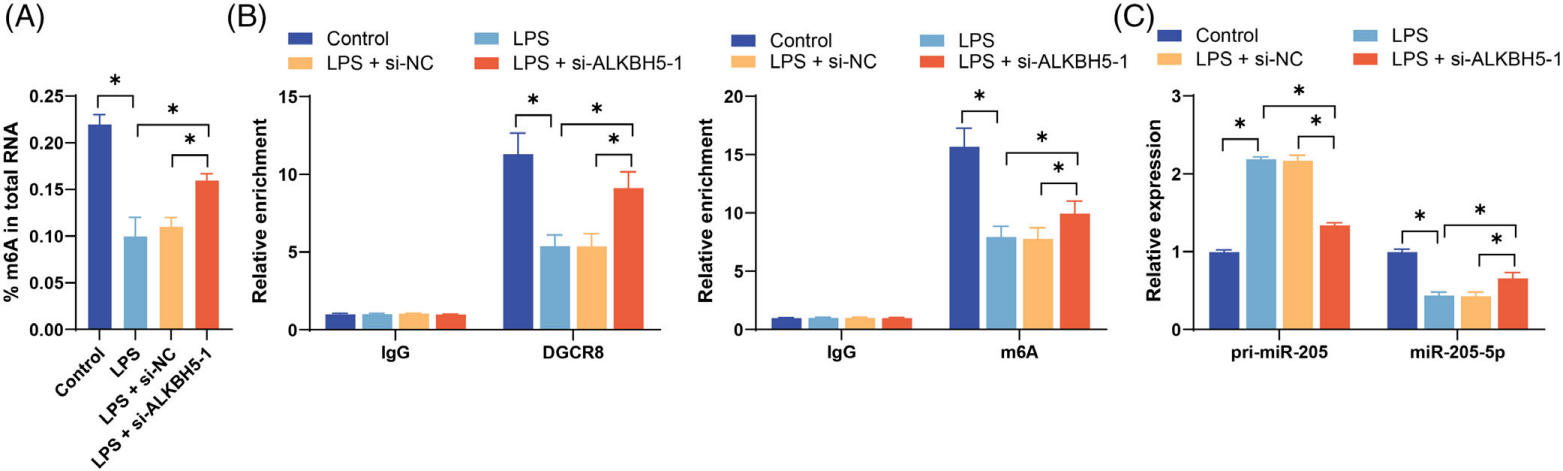
ALKBH5 inhibits the processing of pri‐miR‐205 into miR‐205‐5p by removing m6A modifications. (A) Total RNA m6A was detected with an m6A RNA methylation quantification kit. (B) The levels of DCGR8‐bound and m6A‐modified pri‐miR‐205 were detected by RIP. (C) Pri‐miR‐205 and miR‐205‐5p expression was detected via qRT‐PCR. The cell experiments were independently repeated three times. The data are presented as the mean ± SD. The data in panel A were analyzed via one‐way ANOVA, and the data in panels B and C were analyzed via two‐way ANOVA, followed by Tukey's multiple comparisons test. *p < 0.05. ALKBH5, AlkB homolog 5; miR‐205‐5p, microRNA‐205‐5p; RIP, RNA immunoprecipitation; qRT‐PCR, quantitative real‐time polymerase chain reaction.

ALKBH5 functions as an important m6A demethylase, and its elevated expression leads to a decrease in RNA m6A levels in cells. After the inhibition of ALKBH5, the RNA m6A level in the cells increased (*p* < 0.05, Figure 2A). Previous reports have demonstrated that miR‐205‐5p expression is decreased in SA‐AKI.^15^ We speculate that ALKBH5 inhibits the processing of pri‐miR‐205 into miR‐205‐5p by removing m6A modifications in SA‐AKI. The RIP results revealed that the levels of pri‐miR‐205 bound to DGCR8 and m6A‐modified pri‐miR‐205 decreased after LPS treatment, whereas the inhibition of ALKBH5 reversed these trends (*p* < 0.05, Figure 2B). Moreover, LPS treatment upregulated pri‐miR‐205 expression and downregulated miR‐205‐5p expression, whereas inhibition of ALKBH5 led to the opposite results (*p* < 0.05, Figure 2C). These results indicate that ALKBH5 inhibits the processing of pri‐miR‐205 into miR‐205‐5p by removing m6A modifications.

### 3.3 Inhibition of miR‐205‐5p partially reverses the inhibitory effect of ALKBH5 silencing on LPS‐induced EMT in cells

**FIGURE 3:**
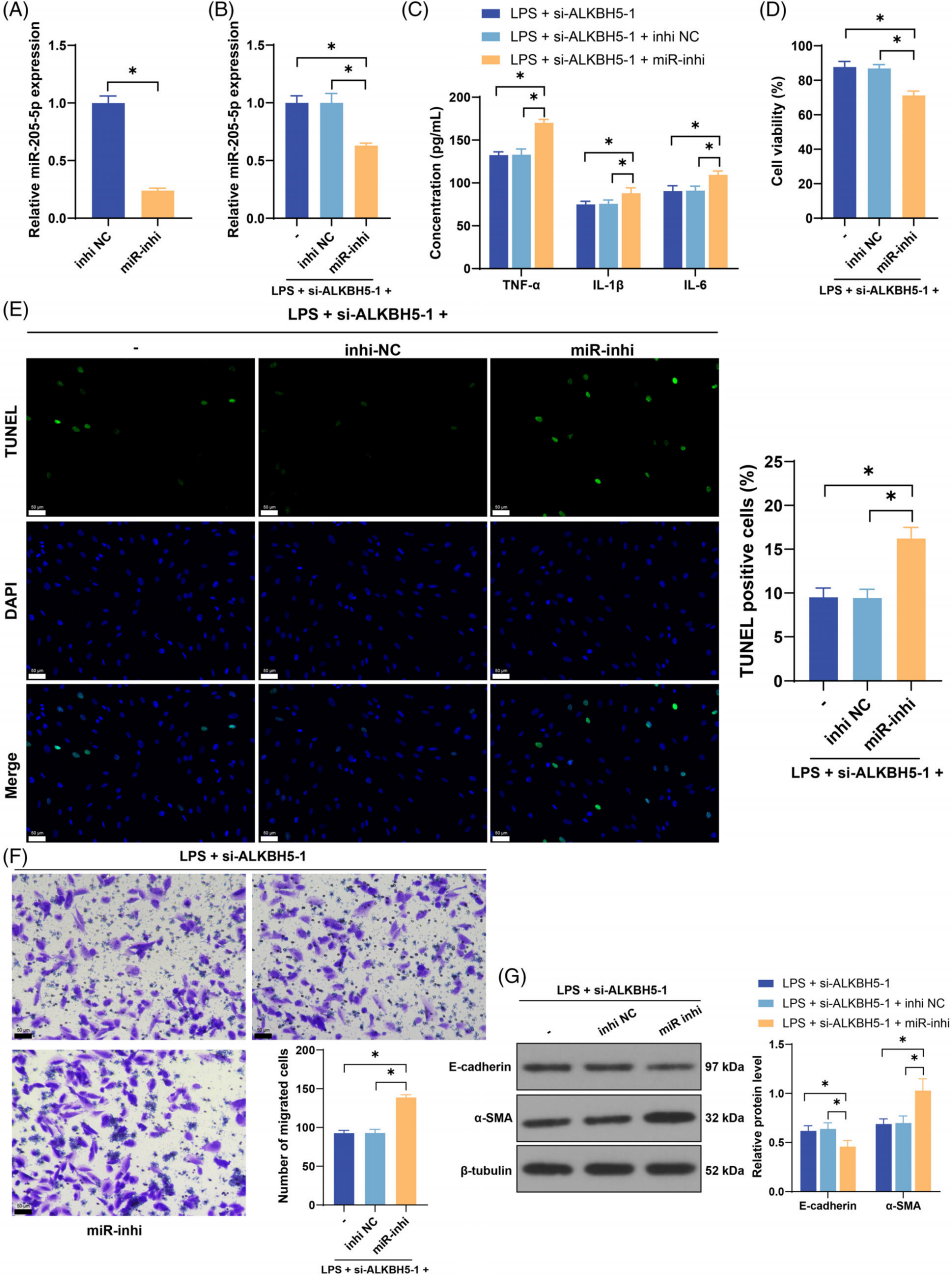
Inhibition of miR‐205‐5p partially reverses the inhibitory effect of ALKBH5 silencing on LPS‐induced EMT in cells. A miR‐205‐5p inhibitor (miR‐inhi) was transfected into HK‐2 cells, with an inhibitor NC (inhi NC) used as a control. (A) Transfection efficiency was confirmed by qRT‐PCR. Then, the cells were subjected to combined treatment with si‐ALKBH5‐1. (B) MiR‐205‐5p expression was detected via qRT‐PCR. (C) The levels of TNF‐α, IL‐1β, and IL‐6 in cells were detected via ELISA. (D) Cell viability was measured by a CCK‐8 assay. (E) Cell apoptosis was evaluated by TUNEL staining. (F) Cell migration was measured via Transwell assays. (G) The protein levels of ALKBH5, E‐cadherin, and α‐SMA were detected by Western blot. The cell experiments were independently repeated three times. The data are presented as the mean ± SD. The data in panel A were analyzed via a *t*‐test. The data in panels B and D–F were analyzed via one‐way ANOVA, and the data in panels C and G were analyzed via two‐way ANOVA, followed by Tukey's multiple comparisons test. *p < 0.05. ALKBH5, AlkB homolog 5; ELISA, enzyme‐linked immunosorbent assay; EMT, epithelial–mesenchymal transition; LPS, lipopolysaccharide; miR‐205‐5p, microRNA‐205‐5p; NC, negative control; qRT‐PCR, quantitative real‐time polymerase chain reaction.

To verify the above mechanism, we inhibited miR‐205‐5p expression in cells (*p* < 0.05, Figure 3A,B) and conducted a combined experiment with si‐ALKBH5‐1. Compared with si‐ALKBH5‐1 transfection alone, the combined treatment resulted in increased levels of proinflammatory factors (*p* < 0.05, Figure 3C), decreased cell viability (*p* < 0.05, Figure 3D), increased apoptosis (*p* < 0.05, Figure 3E), increased cell migration (*p* < 0.05, Figure 3F), decreased E‐cadherin protein levels, and increased α‐SMA protein levels (*p* < 0.05, Figure 3G), which confirmed that ALKBH5 inhibited miR‐205‐5p expression by removing m6A modification, thereby promoting LPS‐induced EMT.

### 3.4 miR‐205‐5p targets DDX5 expression

**FIGURE 4:**
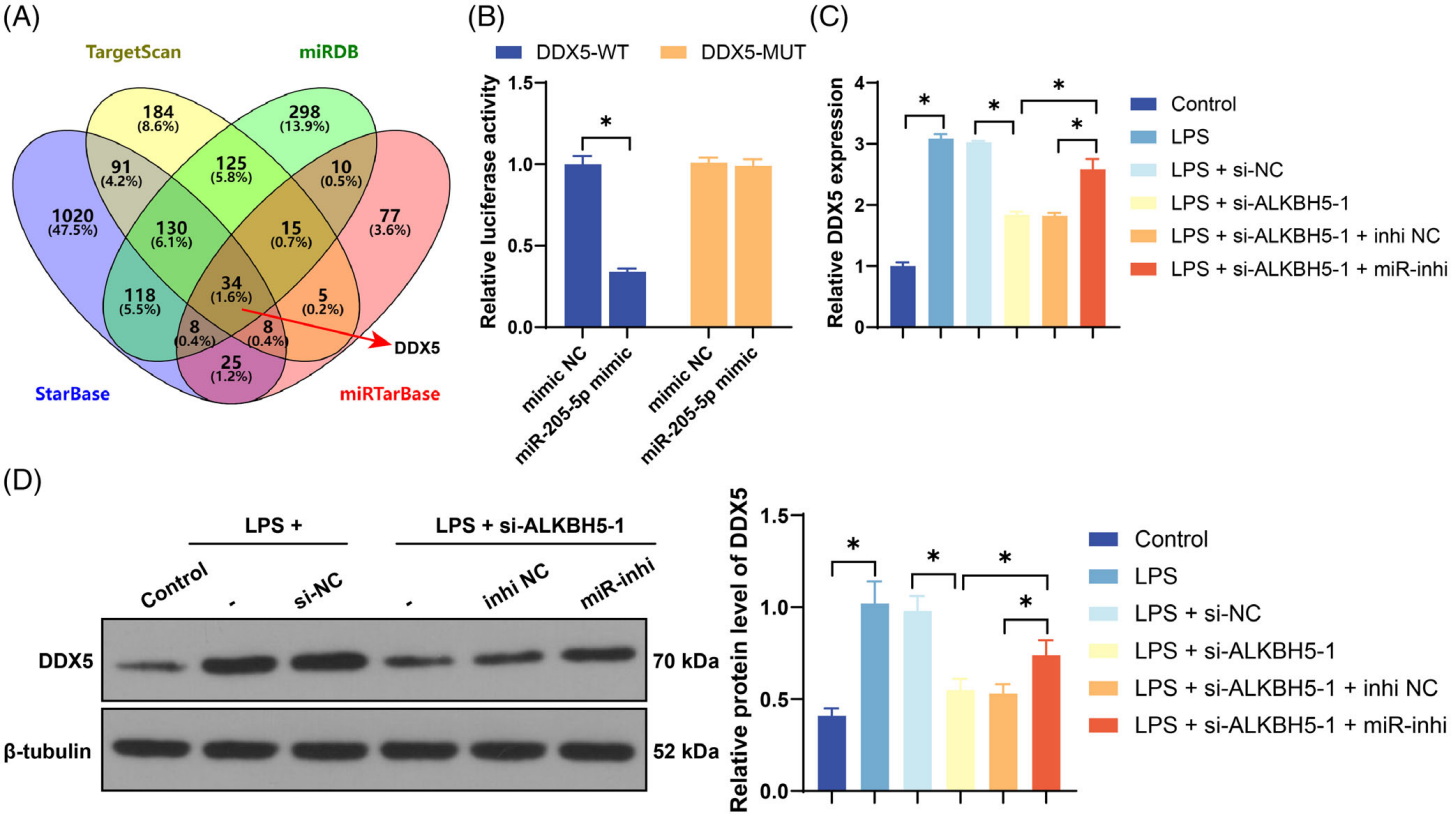
miR‐205‐5p targets DDX5 expression. (A) The target genes downstream of miR‐205‐5p were predicted via the StarBase, TargetScan, miRDB, and miRTarBase databases. (B) The binding between miR‐205‐5p and the 3′‐UTR of DDX5 was verified via a dual‐luciferase assay. (C) DDX5 expression was detected via qRT‐PCR. (D) The DDX5 protein level was detected by Western blotting. The cell experiments were independently repeated three times. The data are presented as the mean ± SD. The data in panel B were analyzed via two‐way ANOVA, and the data in panels C and D were analyzed via one‐way ANOVA, followed by Tukey's multiple comparisons test. *p < 0.05. DDX5, DEAD‐box protein 5; miR‐205‐5p, microRNA‐205‐5p; qRT‐PCR, quantitative real‐time polymerase chain reaction.

We subsequently predicted the target genes downstream of miR‐205‐5p via the StarBase, TargetScan, miRDB, and miRTarBase databases (Figure 4A). DDX5 has been reported to be upregulated in SA‐AKI.^24^ Our dual‐luciferase assay verified that miR‐205‐5p had a target binding relationship with DDX5 (*p* < 0.05, Figure 4B). LPS induced an increase in DDX5 expression, while inhibition of ALKBH5 decreased DDX5 expression, and DDX5 expression was increased in the LPS + si‐ALKBH5‐1 + miR‐inhi group (*p* < 0.05, Figure 4C,D). These results suggest that ALKBH5‐mediated m6A modification inhibits miR‐205‐5p expression to further promote DDX5 expression.

### 3.5 Overexpression of DDX5 partially reversed the inhibitory effect of ALKBH5 silencing on LPS‐induced EMT in cells

**FIGURE 5:**
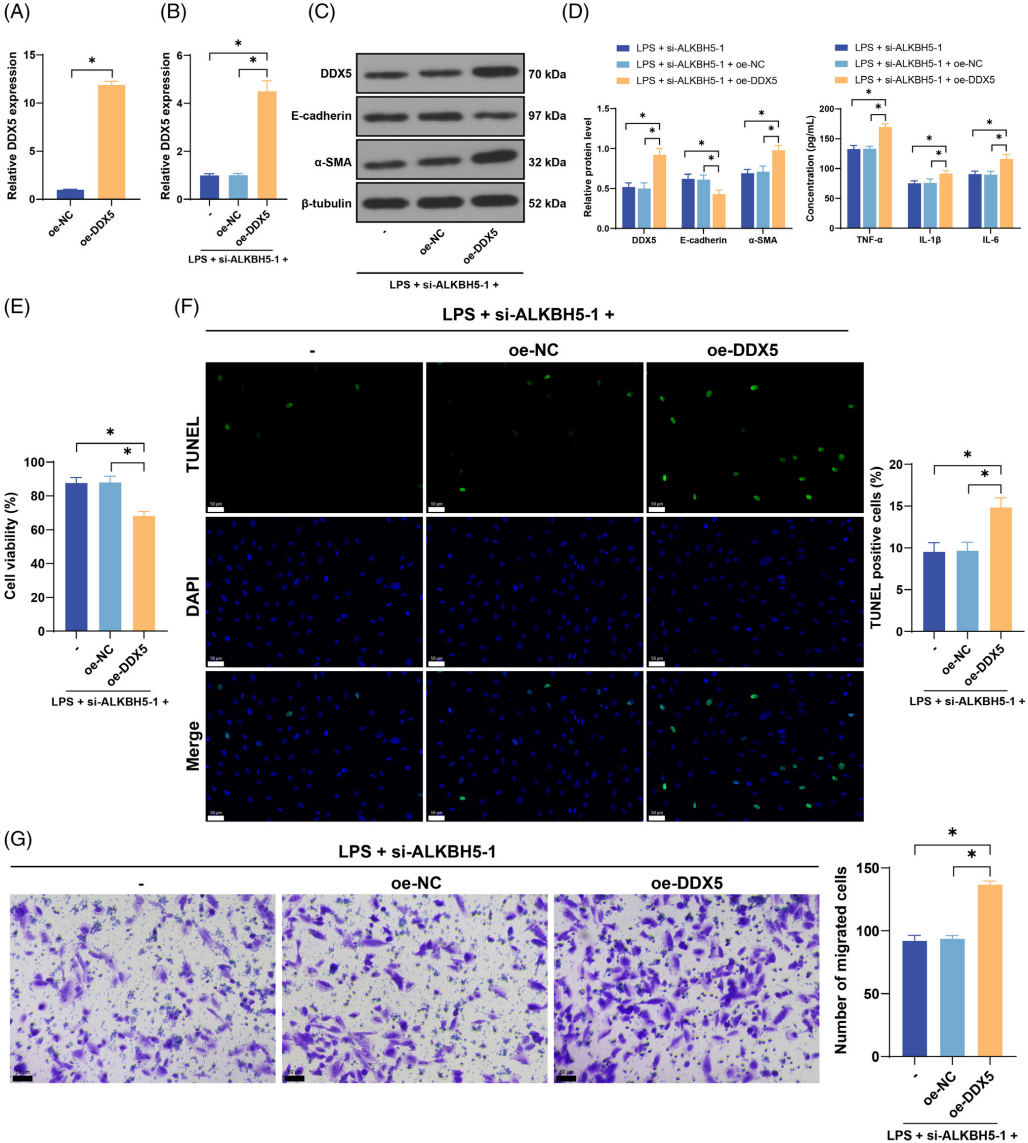
Overexpression of DDX5 partially reversed the inhibitory effect of ALKBH5 silencing on LPS‐induced EMT in cells. A DDX5 overexpression plasmid (oe‐DDX5) was transfected into HK‐2 cells, with an empty plasmid (oe‐NC) used as a control. (A) Transfection efficiency was confirmed by qRT‐PCR. Then, the cells were subjected to combined treatment with si‐ALKBH5‐1. (B) DDX5 expression was detected via qRT‐PCR. (C) The protein levels of ALKBH5, E‐cadherin, and α‐SMA were detected via Western blotting, and the protein levels of E‐cadherin and α‐SMA were detected by Western blot. (D) The levels of TNF‐α, IL‐1β, and IL‐6 in cells were detected via ELISA. (E) Cell viability was measured by a CCK‐8 assay. (F) Cell apoptosis was evaluated by TUNEL staining. (G) Cell migration was measured via Transwell assays. The cell experiments were independently repeated three times. The data are presented as the mean ± SD. The data in panel A were analyzed via a *t*‐test. The data in panels C and D were analyzed via two‐way ANOVA, and the data in panels B and E–G were analyzed via one‐way ANOVA, followed by Tukey's multiple comparisons test. *p < 0.05. ALKBH5, AlkB homolog 5; DDX5, DEAD‐box protein 5; ELISA, enzyme‐linked immunosorbent assay; EMT, epithelial–mesenchymal transition; LPS, lipopolysaccharide; NC, negative control; qRT‐PCR, quantitative real‐time polymerase chain reaction.

Finally, we overexpressed DDX5 in cells (*p* < 0.05, Figure 5A–C) and combined oe‐DDX5 with si‐ALKBH5‐1 for functional rescue experiments. Compared with si‐ALKBH5‐1 transfection alone, the combined treatment resulted in increased levels of proinflammatory factors (*p* < 0.05, Figure 5D), weakened cell viability (*p* < 0.05, Figure 5E), enhanced apoptosis (*p* < 0.05, Figure 5F), increased cell migration ability (*p* < 0.05, Figure 5G), diminished E‐cadherin protein levels, and elevated α‐SMA protein levels (*p* < 0.05, Figure 5C), indicating that ALKBH5 represses miR‐205‐5p expression by removing m6A modification, thereby promoting DDX5 expression and EMT.

## 4 DISCUSSION

**FIGURE 6:**
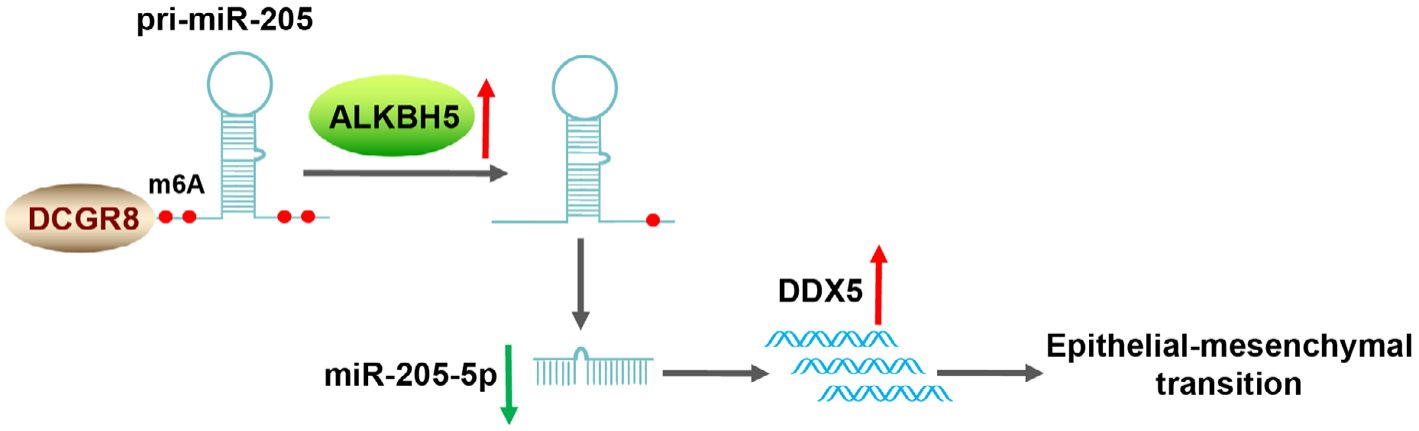
ALKBH5 reduces the binding of DGCR8 to m6A by removing m6A modification, thereby inhibiting the expression of miR‐205‐5p and then upregulating the expression of DDX5 to promote EMT. ALKBH5, AlkB homolog 5; DDX5, DEAD‐box protein 5; EMT, epithelial–mesenchymal transition; miR‐205‐5p, microRNA‐205‐5p.

Despite significant advances in the treatment of sepsis, SA‐AKI remains a severe clinical burden, and further research is warranted to reduce the undesirable consequences.^1^ Compelling evidence has revealed that the EMT process leading to fibrosis of renal tubular epithelial cells is critically associated with the development of AKI.^6^ This study reports for the first time the molecular mechanism by which ALKBH5‐mediated m6A demethylation regulates EMT in SA‐AKI via the miR‐205‐5p/DDX5 axis (Figure 6).

As the most prevalent reversible modification in mammalian mRNAs, m6A methylation is implicated in diverse biological processes associated with kidney injury.^25^ m6A modification affects the progression of EMT and is a potential therapeutic target for treating kidney fibrosis.^26^ In this study, a cell model of SA‐AKI was established in vitro to explore the effect of the m6A demethylase ALKBH5 on LPS‐induced EMT in HK‐2 cells. ALKBH5 depletion decreases cell viability, induces apoptosis, and decreases the levels of inflammatory cytokines in LPS‐stimulated HK‐2 cells, indicating that ALKBH5 is a promising target for the treatment of SA‐AKI.^11^ Knockdown of ALKBH5 can also restrain the malignant potential of clear cell renal cell carcinoma cells by repressing EMT.^27^ Similarly, we detected an increase in ALKBH5 expression in HK‐2 cells under LPS stimulation. LPS treatment resulted in significant upregulation of the expression of proinflammatory factors, attenuation of cell viability, enhancement of apoptosis, a decrease in cell migration, a decrease in the E‐cadherin protein level, and an increase in the α‐SMA protein level, indicating that LPS exacerbates EMT in cells. However, silencing ALKBH5 reversed these trends, indicating that silencing ALKBH5 effectively alleviated LPS‐induced EMT.

Therefore, we explored the specific mechanism of ALKBH5‐mediated demethylation in LPS‐induced EMT. m6A methylation functions as a vital posttranscriptional modification that facilitates the initiation of miRNA biogenesis.^28^ The first step in miRNA biogenesis is the processing of pri‐miRNAs by a microprocessor composed of DGCR8 and DROSHA.^28^ miR‐205‐5p overexpression has been reported to repress LPS‐triggered inflammation and oxidative stress in HK‐2 cells, suggesting a positive role for miR‐205‐5p in slowing SA‐AKI development.^15^ We speculated that ALKBH5 suppressed the processing of pri‐miR‐205 into miR‐205‐5p by removing m6A modifications in SA‐AKI. Under LPS stimulation, the levels of DGCR8‐bound pri‐miR‐205 and m6A‐modified pri‐miR‐205 decreased but then increased after the silencing of ALKBH5. Moreover, LPS upregulated pri‐miR‐205 expression and downregulated miR‐205‐5p expression, while silencing ALKBH5 led to the opposite effects. These findings indicate that ALKBH5 represses the processing of pri‐miR‐205 into miR‐205‐5p by removing m6A modifications. miR‐205‐5p alleviates high‐fat diet‐induced atrial fibrosis^29^ and pulmonary fibrosis in silicosis,^30^ and miR‐205‐5p overexpression can accelerate apoptosis but suppress EMT in renal carcinoma cells.^16^ Consistently, we found that the inhibition of miR‐205‐5p partially abolished the inhibitory effect of ALKBH5 silencing on LPS‐induced EMT.

Furthermore, we predicted the downstream targets of miR‐205‐5p through online databases, among which we focused on DDX5. DDX5 is a prototypical member of the DEAD‐box helicase family and plays a pivotal role in multiple aspects of RNA metabolism.^31^ An increasing number of studies have revealed the functionality of DDX5 in cell cycle regulation, tumorigenesis, and viral infection.^32^ DDX5 is abnormally highly expressed in the pathophysiological process of SA‐AKI.^24^ Our results revealed that LPS induced an increase in DDX5 expression, whereas DDX5 expression was diminished after the inhibition of ALKBH5 but further elevated after combined treatment with the inhibition of miR‐205‐5p. In brief, ALKBH5‐mediated m6A modification decreased miR‐205‐5p expression to further increase DDX5 expression. In LPS‐induced HK‐2 cells, RMRP accelerates apoptosis and exacerbates inflammation by upregulating DDX5.^24^ We found that overexpression of DDX5 counteracted the inhibitory effect of ALKBH5 silencing on LPS‐induced EMT.

## 5 CONCLUSIONS

In summary, ALKBH5 inhibits miR‐205‐5p expression by removing m6A modification to upregulate DDX5 expression, thereby promoting EMT and the AKI‐CKD transition after SA‐AKI. This study also has several limitations. For example, we only carried out relevant tests at the cellular level and did not conduct pathological tests on tissues for mechanism verification. Our findings also need additional animal experimental validation and clinical data support. In future research, we will expand the sample size and combine in vivo and in vitro studies to further explore the mechanism by which ALKBH5‐mediated m6A modification regulates EMT.

## CONFLICT OF INTEREST STATEMENT

The authors declare no conflicts of interest.
